# Dystrophin deficiency exacerbates skeletal muscle pathology in dysferlin-null mice

**DOI:** 10.1186/2044-5040-1-35

**Published:** 2011-12-01

**Authors:** Renzhi Han, Erik P Rader, Jennifer R Levy, Dimple Bansal, Kevin P Campbell

**Affiliations:** 1 Department of Cell and Molecular Physiology, Stritch School of Medicine, Loyola University Medical Center, 2160 S 1st Avenue, Maywood, IL 60558, USA; 2 Department of Molecular Physiology and Biophysics, Howard Hughes Medical Institute, Roy J and Lucille A Carver College of Medicine, The University of Iowa, 285 Newton Road, 4283 CBRB, Iowa City, IA 52242, USA; 3 Department of Neurology, Howard Hughes Medical Institute, Roy J and Lucille A Carver College of Medicine, The University of Iowa, 285 Newton Road, 4283 CBRB, Iowa City, IA 52242, USA; 4 Department of Internal Medicine, Howard Hughes Medical Institute, Roy J and Lucille A Carver College of Medicine, The University of Iowa, 285 Newton Road, 4283 CBRB, Iowa City, IA 52242, USA

**Keywords:** dysferlin, dystrophin, membrane repair, sarcolemmal integrity

## Abstract

**Background:**

Mutations in the genes coding for either dystrophin or dysferlin cause distinct forms of muscular dystrophy. Dystrophin links the cytoskeleton to the sarcolemma through direct interaction with β-dystroglycan. This link extends to the extracellular matrix by β-dystroglycan's interaction with α-dystroglycan, which binds extracellular matrix proteins, including laminin α2, agrin and perlecan, that possess laminin globular domains. The absence of dystrophin disrupts this link, leading to compromised muscle sarcolemmal integrity. Dysferlin, on the other hand, plays an important role in the Ca^2+^-dependent membrane repair of damaged sarcolemma in skeletal muscle. Because dysferlin and dystrophin play different roles in maintaining muscle cell integrity, we hypothesized that disrupting sarcolemmal integrity with dystrophin deficiency would exacerbate the pathology in dysferlin-null mice and allow further characterization of the role of dysferlin in skeletal muscle.

**Methods:**

To test our hypothesis, we generated dystrophin/dysferlin double-knockout (DKO) mice by breeding *mdx *mice with dysferlin-null mice and analyzed the effects of a combined deficiency of dysferlin and dystrophin on muscle pathology and sarcolemmal integrity.

**Results:**

The DKO mice exhibited more severe muscle pathology than either *mdx *mice or dysferlin-null mice, and, importantly, the onset of the muscle pathology occurred much earlier than it did in dysferlin-deficient mice. The DKO mice showed muscle pathology of various skeletal muscles, including the mandible muscles, as well as a greater number of regenerating muscle fibers, higher serum creatine kinase levels and elevated Evans blue dye uptake into skeletal muscles. Lengthening contractions caused similar force deficits, regardless of dysferlin expression. However, the rate of force recovery within 45 minutes following lengthening contractions was hampered in DKO muscles compared to *mdx *muscles or dysferlin-null muscles, suggesting that dysferlin is required for the initial recovery from lengthening contraction-induced muscle injury of the dystrophin-glycoprotein complex-compromised muscles.

**Conclusions:**

The results of our study suggest that dysferlin-mediated membrane repair helps to limit the dystrophic changes in dystrophin-deficient skeletal muscle. Dystrophin deficiency unmasks the function of dysferlin in membrane repair during lengthening contractions. Dystrophin/dysferlin-deficient mice provide a very useful model with which to evaluate the effectiveness of therapies designed to treat dysferlin deficiency.

## Background

Duchenne muscular dystrophy (DMD) is an X-linked recessive disease affecting approximately 1 in 3, 500 males and is caused by defects in the dystrophin gene [1]. Dystrophin is an integral component of the dystrophin-glycoprotein complex (DGC) and is localized to the inner surface of the plasma membrane [2]. Dystrophin plays an important role in linking the cytoskeleton to the sarcolemma through the direct interactions of its N-terminus with F-actin and its C-terminus with β-dystroglycan [2]. This link is extended to the extracellular matrix (ECM) by α-dystroglycan, which binds to laminin α2, agrin and perlecan with high affinity. The dystrophin-mediated continuous link between the cytoskeleton and the ECM is reported to play an important role in stabilizing the sarcolemmal structure, transmitting force laterally and preventing the expansion of muscle membrane damage during lengthening contraction (LC) [3-8]. In DMD patients and *mdx *mice, which also have a mutation in the dystrophin gene, loss of dystrophin disrupts the link between the cytoskeleton and the ECM, leading to the loss of sarcolemmal integrity. This loss of sarcolemmal integrity eventually results in muscle degeneration, necrosis and fibrosis. As a consequence, DMD patients are confined to a wheelchair in their early teens and die in their early 20s as a result of cardiopulmonary failure [9].

The plasma membrane provides a physical barrier between the extracellular space and the intracellular environment, and maintenance of this barrier is crucial for the survival of any cell. We previously showed that skeletal muscle possesses the ability to repair membrane wounding in a Ca^2+^-dependent manner and that dysferlin plays a critical role in this process [10,11]. Mutations in dysferlin cause limb-girdle muscular dystrophy type 2B [12,13], Miyoshi myopathy [13] and a distal anterior compartment myopathy [14].

Dystrophin deficiency renders the muscle susceptible to contraction-induced sarcolemmal injuries [4-8], whereas dysferlin deficiency results in compromised membrane repair [10,15-19]. Taking into consideration these two different roles in maintaining sarcolemmal integrity, we hypothesized that skeletal muscle membrane stability mediated by the DGC and dysferlin-mediated membrane repair are both essential for the maintenance of muscle membrane integrity and function. On the basis of this hypothesis, we predicted that a combined deficiency in both dysferlin and dystrophin would lead to more severe muscle pathology due both to an increased susceptibility to muscle membrane injuries in the absence of dystrophin and to defective membrane repair in the absence of dysferlin. To test this hypothesis, we generated mice that lack both dysferlin and dystrophin. The dystrophin/dysferlin double-knockout (DKO) mice developed more severe muscle pathology than either *mdx *mice or dysferlin-null mice, which is reflected by the higher number of regenerated muscle fibers, increased serum creatine kinase (CK) levels and more Evans blue dye (EBD) uptake in their muscles. These data show that dysferlin-mediated membrane repair limits the severity of dystrophic changes in *mdx *skeletal muscle.

## Methods

### Mice

The DKO mice were generated by breeding female *mdx*/C57BL/10ScSn (*mdx*) mice with male dysferlin-null mice [10] through two generations (Additional file 1, Figure S1). F2 pups were genotyped using recently improved methods for genotyping the dysferlin allele [20] and the *mdx *allele [21] to identify the double-mutant mice. The genetic background of DKO mice is a mixture of C57/BL6, 129/SVJ and C57BL/10ScSn. The wild-type (WT) littermates with the same genetic background were used as controls. Mice were maintained at The University of Iowa Animal Care Unit and treated in accordance with animal use guidelines. All animal studies were authorized by the Animal Care, Use, and Review Committee of The University of Iowa.

### Serum creatine kinase assay

Using a Microvette CB 300 (Sarstedt AG & Co, Newton, NC), we collected blood required for quantitative, kinetic determination of serum CK activity by mouse tail vein bleeds from nonanesthetized, restrained mice. Red blood cells were pelleted by centrifugation at 10, 000 rpm for 4 minutes, and serum was separated, collected and analyzed immediately without freezing. Serum CK assays were performed with an enzyme-coupled assay reagent kit (Stanbio Laboratory, Boerne, TX, USA) according to the manufacturer's instructions. Absorbance at 340 nm was measured every 30 seconds for 2 minutes at 37°C so that changes in enzyme activity could be calculated.

### Histological and immunofluorescence analyses

Muscles (masseter, quadriceps, hamstrings, gluteus, gastrocnemius, tibialis anterior, iliopsoas and diaphragm) were dissected and frozen in isopentane cooled to -165°C in liquid nitrogen. Seven-micron cryosections were cut and fixed in 10% neutral buffer formalin for five minutes. After fixation, the slides were washed for five minutes under running water followed by H & E staining (Surgipath Medical Industries, Inc/Leica Microsystems, Richmond, IL, USA). H & E-stained sections were analyzed by light microscopy (Leica Microsystems Inc, Buffalo Grove, IL, USA; Carl Zeiss Microscopy, LLC, Thornwood, NY, USA). Immunofluorescence analyses were also performed on 7-μm cryosections. Sections were processed for immunofluorescence microscopy and analyzed with an epifluorescence microscope (Leica Microsystems Inc, and Carl Zeiss Microscopy, LLC). Mouse anti-dysferlin (Hamlet-1, Novocastra, Newcastle, UK) mAb and rabbit pAb against β-dystroglycan [22], sarcospan [23], dystrophin (Abcam, Cambridge, MA, USA) and laminin α2 chain (AXXORA LLC, San Diego, CA, USA) were used for immunofluorescence analysis. The central nucleated and total muscle fibers were counted on muscle sections costained with anti-laminin α2 antibody and 4', 6-diamidino-2-phenylindole (Sigma-Aldrich, St Louis, MO, USA) using Image-Pro Plus version 6 software (Media Cybernetics, Inc, Bethesda, MD, USA).

### Western blot analysis

Proteins were extracted from 20 to 35 cryosections (30 μm thick) of quadriceps tissue from each mouse using 250 μl of PBS plus 1% Triton X-100, 0.5% SDS and protease inhibitors. The protein samples in the supernatant were mixed with 80 μl of 5× Laemmli sample buffer, and 70-μl final samples were resolved by SDS-PAGE on 3% to 15% linear gradient gels and transferred onto polyvinylidene fluoride Immobilon-FL membrane (Millipore, Billerica, MA, USA). The membranes were blocked with fish gelatin in Tris-buffered saline (TBS) and incubated with primary antibodies (mouse anti-dysferlin mAb Hamlet, mouse anti-β-dystroglycan 8D5 mAb, mouse anti-dystrophin mAb and rabbit anti-dihydropyridine receptor (anti-DHPR) α2 pAb [24]). Blots were washed with TBS + 0.1% Tween 20 and incubated with infrared dye-conjugated secondary antibodies (Pierce Biotechnology/Thermo Fisher Scientific, Inc, Rockford, IL, USA). After washing, blots were captured using the Odyssey Imaging System (LI-COR, Lincoln, NE, USA).

### Evans blue dye uptake

Evans blue dye (Sigma-Aldrich) was dissolved in PBS (10 mg/ml) and sterilized through a 0.2-μm pore size filter. The mice were anesthetized by ketamine injection (0.1 ml/10 g body weight), and 0.05 ml/10 g body weight dye solution was injected intraperitoneally. The animals were killed 24 hours after injection, and their skeletal muscles were isolated and frozen in isopentane cooled to -165°C in liquid nitrogen. Microscopic evaluation of EBD uptake was performed on 7-μm muscle cryosections. Muscle cryosections were fixed in cold acetone at -20°C for 10 minutes, washed with PBS, coverslipped in VECTASHIELD Mounting Medium (Vector Laboratories, Burlingame, CA, USA) and evaluated by fluorescence microscopy.

### Force measurement

Contractile properties were measured *in vitro *on extensor digitorum longus (EDL) muscles from WT, dysferlin-null, *mdx *and DKO mice (12 to 22 weeks of age) as described previously [3,25]. The mice were anesthetized with an intraperitoneal injection of 2% avertin (0.0015 ml/g body weight), and thoracotomy was performed. EDL muscles were immersed in an oxygenated bath (95% O_2_, 5% CO_2_) that contained Ringer's solution (pH 7.4) at 25°C. For each muscle, one end tendon was tied securely with a 6-0 suture to a dual-mode servomotor (Aurora Scientific, Inc, Aurora, ON, Canada) and the other tendon was clamped to a fixed post. Using twitches with a pulse duration of 0.2 milliseconds, the voltage or current of stimulation was increased to achieve a maximum twitch and then increased slightly. Twitches were then used to adjust the muscle length to the optimum length for force development (L_o_). Fiber length (L_f_) was determined by multiplying L_o _by the ratio of fiber length to muscle length (0.45) [3,25,26]. The muscle length was set at L_o_, and EDL muscles were stimulated for 300 milliseconds. The stimulation frequency was increased until the force reached a plateau at maximum isometric tetanic force (P_0_). The susceptibility to LC-induced injury was assessed by subjecting each muscle to eight LCs at a rate of one LC every three minutes [3,25,26]. Each LC consisted of maximally activating the muscle at a fixed length for 100 milliseconds, then stretching the muscle at a strain of 30% of L_f _at a strain a velocity of 1 L_f_/second. Muscle activation ceased upon achieving the 30% strain and was returned to L_o _at the same velocity. To assess the force deficit generated by this assay, a measurement of P_0 _was taken three minutes after the last LC and repeated at 15, 30 and 45 minutes. The total fiber cross-sectional area and specific P_0 _(kN/m^2^) were calculated based on measurements of muscle mass, L_f _and P_0_. A generalized linear model with repeated-measures analysis of variance (ANOVA) using SPSS software (SPSS, Inc, Chicago, IL, USA) was used to determine whether time after LC, dysferlin and dystrophin were significant factors.

### Statistics

Data were calculated according to analysis of variance (ANOVA) and are expressed as means ± SEM. Where appropriate, the significance of differences between multiple mouse models was assessed using one-way ANOVA with Student-Newman-Keuls posttests, and the significance of differences between two experimental groups were assessed using an unpaired two-tailed Student's *t*-test. *P *< 0.05 was accepted as significant.

## Results

### Disrupted dystrophin and dysferlin expression in skeletal muscles of DKO mice

**Figure 1:**
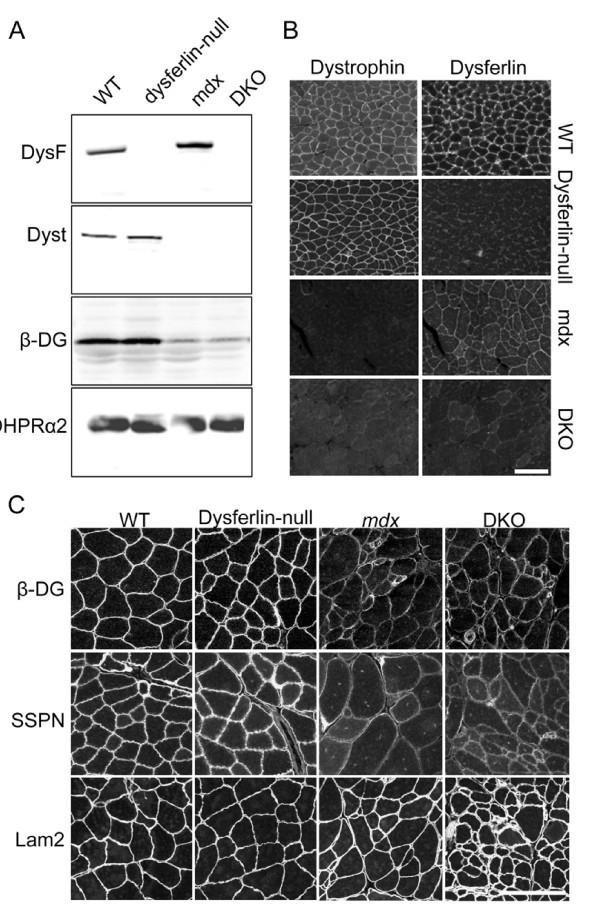
**Disrupted expression of dysferlin and dystrophin in skeletal muscle of dystrophin/dysferlin double-knockout mice**. **(A) **Western blot showing the expression pattern of dysferlin (DysF), dystrophin (Dyst), β-dystroglycan (β-DG) and dihydropyridine receptor α2 (DHPRα2) in skeletal muscle tissue lysates from wild-type (WT), dysferlin-null, *mdx *and dystrophin/dysferlin double-knockout (DKO) mice. **(B) **Expression of dystrophin and dysferlin in skeletal muscles from WT, dysferlin-null, *mdx *and DKO mice were examined by immunofluorescence staining. **(C) **β-DG and sarcospan (SSPN) were greatly diminished at the sarcolemma of *mdx *and DKO muscles, but laminin α2 (Lam2) staining was not reduced. Scale bars: 100 μm.

Both immunofluorescence and Western blot analyses confirmed the complete loss of both dystrophin and dysferlin in the skeletal muscle membranes of the DKO mice (Figures 1A and 1B) [15]. Mutations in dystrophin disrupt the stability of the entire DGC at the sarcolemma, which in turn renders the muscle susceptible to contraction-induced injuries [2]. To assess the expression of DGC components in DKO muscle, we performed immunofluorescence and immunoblot experiments using the specific antibodies against β-dystroglycan and sarcospan, two DGC components. The WT and dysferlin-null muscles showed normal expression of β-dystroglycan and sarcospan (Figures 1A and 1C), suggesting a stable DGC at the sarcolemma. The *mdx *and DKO muscles showed a reduction in the expression of both proteins (Figures 1A and 1C), suggesting a disrupted DGC in the muscles of these mice. The expression levels of non-DGC proteins DHPR α2 and laminin α2 were also examined, and we found that they were not reduced in the DKO muscles (Figures 1A and 1C).

### Increased muscle histopathology in the DKO mice

**Figure 2:**
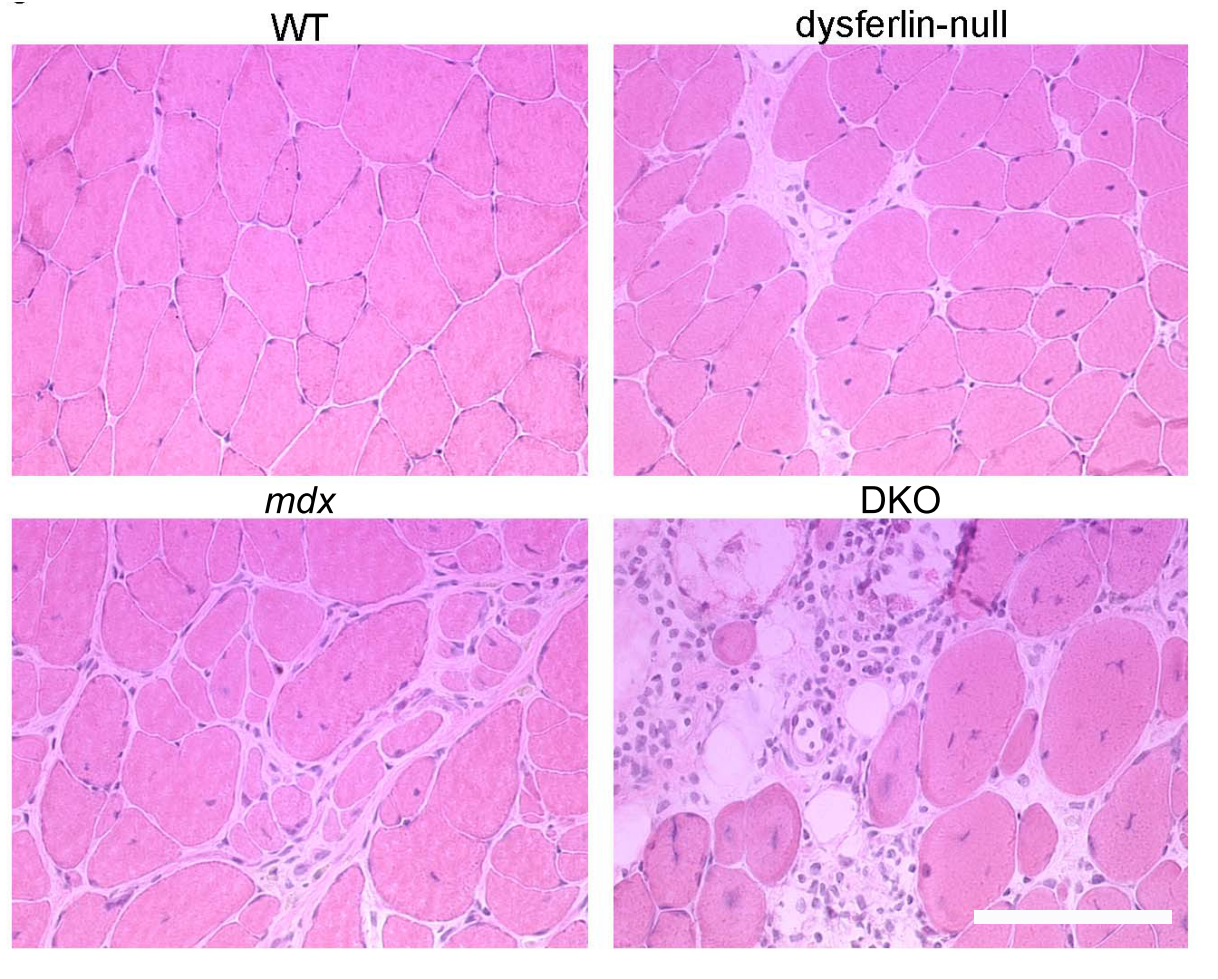
**H & E staining of masseter muscle sections from WT, dysferlin-null, *mdx *and DKO mice at six months of age**. Scale bar: 100 μm.

Defects in either dysferlin or dystrophin lead to deficits in skeletal muscle [1,12,13]. Therefore, we carefully examined the function and histopathology of skeletal muscles in the DKO mice. We observed that many of the DKO mice developed malocclusion. Histological examination of the DKO mandible muscles by H & E staining revealed greater dystrophic features than either the *mdx *or dysferlin-null masseter muscles (Figure 2). Because rodent incisors grow continuously from birth and are kept worn down and sharp by continuously gnawing [27], the more severe muscle pathology of the mandibular muscles in DKO mice suggests increased muscle weakness of these muscles as a reason for the long incisor growth. The front teeth of mice with malocclusion were regularly clipped, allowing them access to and ingestion of food.

**Figure 3:**
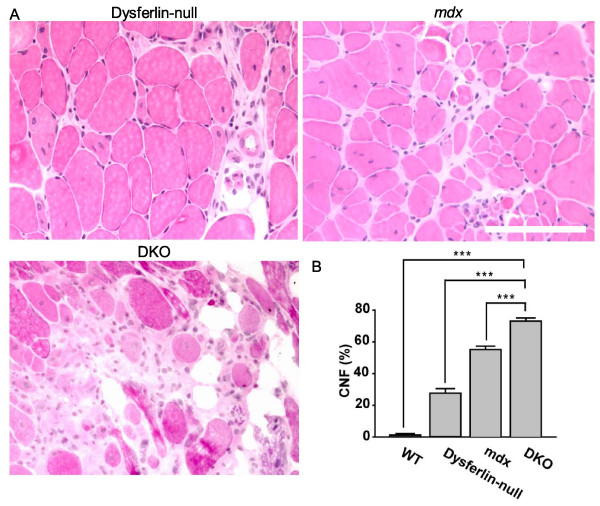
**Histopathological analyses of quadriceps muscle sections from DKO mice**. **(A) **H & E-stained quadriceps muscle sections from dysferlin-null, *mdx *and DKO mice at six months of age. Scale bar: 100 μm. **(B) **Quantitative analysis of centrally nucleated muscle fibers (CNF) in quadriceps muscles from WT, dysferlin-null, *mdx *and DKO mice (*n *= 6 per group) at six months of age. Each group was significantly different from all the other groups. For clarity, significance is shown only for the comparisons with the DKO mice. ***P < 0.001.

The quadriceps muscle sections were also analyzed at six months of age in DKO mice and compared with the muscles isolated from age-matched controls, dysferlin-null and *mdx *mice. The DKO quadriceps muscle tissues exhibited more severe muscular dystrophic pathology compared with dysferlin-null and *mdx *mice (Figure 3). Centrally nucleated muscle fibers, which indicate regenerating fibers, were quantified in quadriceps muscles from six-month-old WT, dysferlin-null, *mdx *and DKO mice. Of the total muscle fibers, 28% ± 3% in dysferlin-null mice, 55% ± 2% in *mdx *mice and 73% ± 2% in DKO mice were centrally nucleated compared to 1.3% ± 0.2% in WT mice (six mice per group) (Figure 3B). Muscle histopathology of aged DKO mice was also performed, including the triceps, gastrocnemius, diaphragm, tibialis anterior, iliopsoas, hamstring and gluteus muscles (Additional file 2, Figure S2). All muscles from DKO mice exhibited severe pathology, including widespread muscle necrosis, fibrosis and fatty replacement. These results are consistent with our hypothesis that dystrophin and dysferlin play nonredundant roles in maintaining muscle function.

### Severely compromised muscle sarcolemmal integrity in the DKO mice

**Figure 4:**
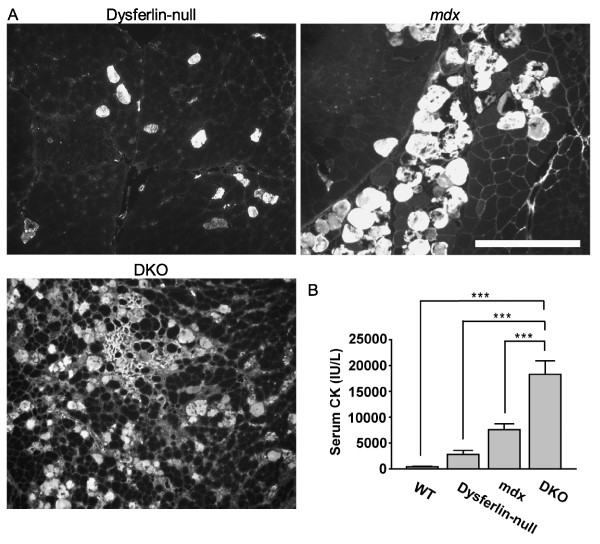
**Evans blue dye uptake analyses of quadriceps muscles and serum CK measurements**. **(A) **Evans blue dye (EBD) fluorescence photomicrographs of quadriceps muscle sections from dysferlin-null, *mdx *and DKO mice at six months of age. Scale bar: 100 μm. **(B) **Serum creatine kinase (CK) levels were significantly different (*P *< 0.001) between DKO mice (*n *= 6) and the other groups (*n *= 5, 4 and 6 for WT, dysferlin-null and *mdx *mice, respectively). Values for *mdx *mice were significantly different from those for WT mice (*P *= 0.018). For clarity, significance is shown only for the comparisons with the DKO mice. ***P < 0.001.

To examine the sarcolemmal integrity of the skeletal muscle fibers in the DKO mice, we injected EBD and analyzed its uptake into the quadriceps skeletal muscles. EBD is a membrane-impermeable dye that binds serum albumin. A cell can uptake this dye only if the plasma membrane of the cell is compromised. Therefore, the presence of EBD-positive fibers indicates the presence of membrane disruptions in muscle fibers. Individual EBD-positive muscle fibers were scattered throughout the muscle sections of dysferlin-null mice, and the muscle sections of *mdx *mice showed clusters of EBD-positive fibers [28] (Figure 4A). Interestingly, the DKO mice showed both patterns of dye uptake: individual and clusters of EBD-positive muscle fibers (Figure 4A).

Membrane disruptions and muscle damage not only allow EBD uptake but also lead to leakage of cytosolic contents such as CK, which can then be detected in the serum. Measurements of serum CK levels provide an index of the active skeletal muscle necrosis and the presence of membrane disruptions. Our analysis revealed that the DKO mice had serum CK levels twofold higher than those of *mdx *mice and tenfold higher than those of dysferlin-null mice (Figure 4B). Taken together, these data suggest that the dystrophin and dysferlin double-deficiency results in decreased sarcolemmal integrity.

### Severe functional deficits in the DKO muscles

**Figure 5:**
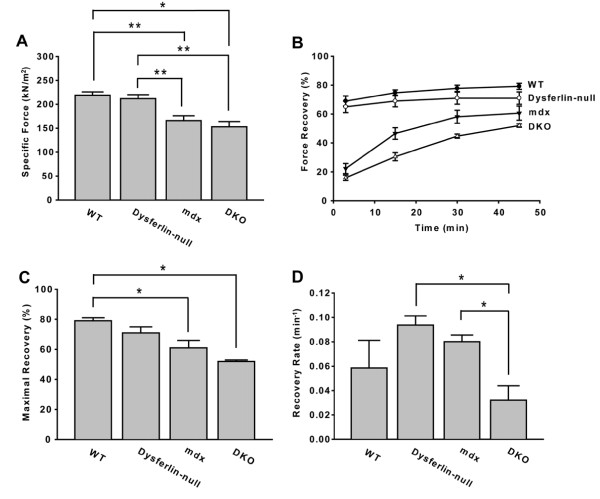
**Effects of dysferlin and dystrophin deficiencies on skeletal muscle contractile properties**. We investigated the extensor digitorum longus (EDL) muscles taken from WT (*n *= 4), dysferlin-null (*n *= 6), *mdx *(*n *= 8) and DKO mice (*n *= 3). **(A) **Tetanic force measurements prior to LC. *P < 0.05 and **P < 0.01. **(B) **Tetanic force recovery following LC. Dysferlin deficiency had a significant effect on force recovery in the absence of dystrophin, as indicated by a significant interaction (*P *= 0.04) between dysferlin, dystrophin and time after LC on the basis of analysis of variance. **(C) **Summary of estimated maximal recovery levels following LC. *P < 0.05. **(D) **Summary of force recovery rate following LC. *P < 0.05.

To examine whether a double-deficiency of dysferlin and dystrophin also leads to a more severe functional disturbance, we measured the force production of EDL muscles and their responses to LCs. The specific forces were 219 ± 7 kN/m^2 ^for WT muscle and 212 ± 8 kN/m^2 ^for dysferlin-null muscle, suggesting that the absence of dysferlin alone does not lead to a deficit of specific force. The specific forces for *mdx *mice (166 ± 8 kN/m^2^) were significantly lower than those for WT mice. The absence of dysferlin had no effect in the *mdx *background, as indicated by a lack of significant difference in specific forces of DKO muscles (153 ± 11 kN/m^2^) compared to those of *mdx *muscles (Figure 5A). However, dysferlin had a significant role in the force recovery following LCs, particularly in the context of an *mdx *background (Figure 5B). Both the WT and dysferlin-null muscles recovered marginally within 45 minutes post-LC (WT: 69% ± 4% at 3 minutes vs 79% ± 2% at 45 minutes; dysferlin-null: 65% ± 4% at 3 minutes vs 71% ± 4% at 45 minutes). Although the force in *mdx *muscles was greatly diminished at 3 minutes post-LC, it gradually recovered from 22% ± 4% at 3 minutes to 61% ± 5% at 45 minutes (Figure 5B). Interestingly, the DKO muscles also recovered significantly from 16% ± 2% at 3 minutes to 52% ± 1% at 45 minutes (Figure 5B). However, dysferlin deficiency significantly slowed the recovery of force in the absence of dystrophin (Figure 5B). To estimate the maximal recovery level and the recovery rate, we fitted the data to a one-phase association equation. Particularly, we found that the recovery rate in DKO muscles (0.032 ± 0.012/minute) was significantly lower than in *mdx *muscles (0.080 ± 0.006/minute), although there was no difference in the maximum recovery levels (Figures 5C and 5D). These data suggest that dysferlin deficiency impairs the force recovery following LCs in *mdx *mice, indicating that compromised membrane repair [10] has consequences for post-LC force generation.

## Discussion

Skeletal muscle is associated with significant stress and strain during muscle contraction. Previous studies have shown at least two mechanisms that skeletal muscle cells utilize to maintain sarcolemmal integrity: a tightly associated basal lamina mediated by the dystroglycan complex limits the extent of plasma membrane damage [3] and membrane repair machinery involving multiple proteins that actively restore membrane integrity following limited levels of membrane disruption, such as dysferlin [10,15,18]; calpain [29]; annexins A1, A2 and A5 [18,30,31]; and MG53 [32,33]. A defect in either mechanism is detrimental to the muscle, as manifested by the fact that genetic mutations in either the DGC components (for review, see [34]) or dysferlin [12-14] cause various muscular dystrophies. In the present work using mouse models, we have further demonstrated that a double-deficiency in both dystrophin and dysferlin results in more severe muscular dystrophy. The results of our work are in agreement with those of Shaw *et al*. [35], who showed that mice overexpressing the Coxsackie virus and adenovirus receptor transgene demonstrate decreased expression levels of dystrophin and dysferlin, which may lead to an associated myopathy. Our work provides genetic evidence that dysferlin and the DGC are independent pathways for the maintenance of sarcolemmal integrity in muscle and further highlights the importance of these two pathways in muscle health.

Contraction-induced injury is characterized by two distinct phases: an initial injury and a delayed secondary injury from the inflammatory response. The initial injury consists of mechanical disruption of sarcomeres followed by impaired excitation-contraction coupling and Ca^2+ ^signaling and finally by activation of Ca^2+^-sensitive degradation pathways [36]. Interestingly, for muscle with an intact DGC, whether membrane damage occurs during the initial injury phase is unclear; but if it exists, it is dysferlin-independent [25,37,38]. Several hours to days following the initial injury, infiltration of inflammatory factors damage muscle fibers and release reactive oxygen species (ROS), which aid in clearing disrupted myofibrils but also damage previously undamaged myofibrils. At this stage, ROS potentially cause damage to the sarcolemma, possibly through lipid peroxidation [39,40]. Investigators in previous studies have demonstrated that dysferlin is important in limiting the extent of secondary injury. For example, three days following a protocol of small strain LCs, muscles of dysferlin-deficient mice displayed increased macrophage infiltration, decreased ability to seal off a membrane-impermeable dye and increased force deficits [38,41]. These data indicate that defects in membrane repair or alteration in repair result in chemoattraction of immune cells [38,41].

Previously, we demonstrated that a combined deficiency in dysferlin and dystrophin results in the development of a pronounced early-onset cardiomyopathy resembling that seen in DMD patients, in contrast to a mild, slowly progressing cardiac manifestation in *mdx *mice [15]. Thus the DKO mouse model is well-suited to the study of the molecular mechanisms of and therapy for cardiomyopathy in DMD. Our present study reveals for the first time that dystrophin deficiency unmasks a role for dysferlin in repairing membrane damage during the initial injury elicited by LCs. Compared to WT skeletal muscle, *mdx *muscle had a much greater force deficit measured three minutes after LC (78% in *mdx *vs 31% in WT). However, by 45 minutes, the force in *mdx *muscles recovered from 22% to over 60% of the preinjury level, whereas the WT and dysferlin-null muscles gained only 10% (from 69% to 79%) and 6% (from 65% to 71%), respectively. These data suggest that, unlike WT and dysferlin-null muscle, in which LCs induce little membrane damage, *mdx *muscle undergoes more severe membrane damage, which requires an active membrane repair process to restore membrane integrity. DKO muscles are also repair-competent, as the force also recovered from 16% at 3 minutes to 52% at 45 minutes. However, by comparing the recovery rate constants of *mdx *muscles and DKO muscles, we found that the recovery rate of the DKO muscles was less than half the rate of *mdx *muscles. Therefore, our data derived from using a LC assay reveal that dystrophin deficiency sufficiently unmasks the membrane repair role of dysferlin. The DKO mouse would be a very useful model with which to determine the efficacy of a therapeutic treatment designed for dysferlinopathy.

## Conclusion

Taken together, our results show that both DGC-mediated membrane stability and dysferlin-mediated membrane repair contribute to the function and maintenance of skeletal muscle. The studies presented here suggest that the DKO mouse model may be a valuable tool in the development of therapies designed to treat dysferlinopathies.

## Abbreviations

CK: creatine kinase; DGC: dystrophin-glycoprotein complex; DHPR: dihydropyridine receptor; DKO: dystrophin/dysferlin double-knockout; DMD: Duchenne muscular dystrophy; EBD: Evans blue dye; ECM: extracellular matrix; EDL: extensor digitorum longus; H & E: hematoxylin and eosin; LC: lengthening contraction; mAb: monoclonal antibody; pAb: polyclonal antibody; PBS: phosphate-buffered saline; ROS: reactive oxygen species.

## Competing interests

The authors declare that they have no competing interests.

## Authors' contributions

RH conceived the study, carried out the histopathological studies, participated in the sequence alignment and drafted the manuscript. ER carried out the force measurement experiments and performed the statistical analysis. JL carried out the immunoassays. DB initiated the mouse breeding and participated in sequence alignment. KC conceived the study, participated in its design and coordination and helped to draft the manuscript. All authors read and approved the final manuscript.

## Supplementary Material

Additional file 1 **Figure S1 Breeding strategy to generate dystrophin/dysferlin double-knockout mice**. Male dysferlin-null mice were mated with *mdx *female mice, then F1 heterozygous males and females were bred to generate F2 males and females. Of the F2 males and females, 12.5% are predicted to be DKO mice. Click here for file

Additional file 2 **Figure S2 Histopathology analyses of various muscles from dystrophin/dysferlin double-knockout mice**. H & E-stained muscle sections of triceps (TC), gastrocnemius (GA), diaphragm (DIA), tibialis anterior (TA), iliopsoas (IP), hamstring (HS) and gluteus (GT) muscles from dystrophin/dysferlin double-knockout (DKO) mice at one and one-half years of age. Scale bar: 100 μm. Click here for file
