# Supplementary figures and images for: Dystrophin deficiency exacerbates skeletal muscle pathology in dysferlin-null mice
Source: Skelet Muscle. 2011 Dec 1;1:35. doi: 10.1186/2044-5040-1-35 (PMC3287108; doi:10.1186/2044-5040-1-35)

Figure S1

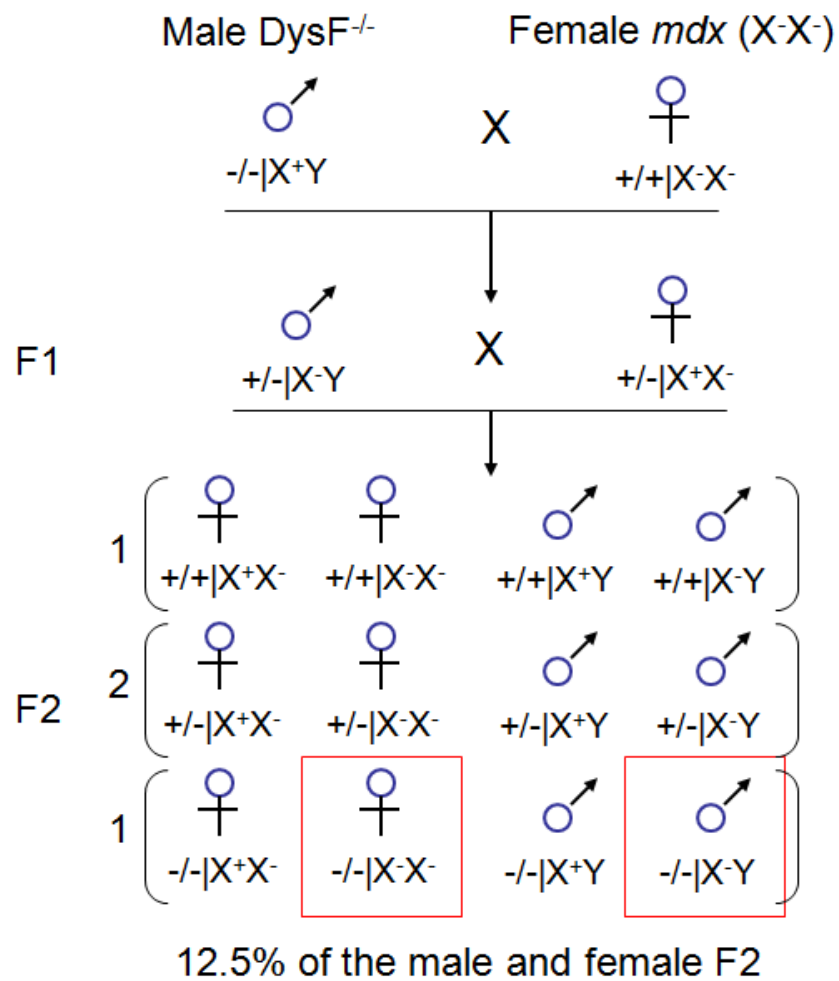

Supplement: Additional file 1 — Figure S1 Breeding strategy to generate dystrophin/dysferlin double-knockout mice. Male dysferlin-null mice were mated with mdx female mice, then F1 heterozygous males and females were bred to generate F2 males and females. Of the F2 males and females, 12.5% are predicted to be DKO mice. [file 2044-5040-1-35-S1.PDF]

Figure S2

TC

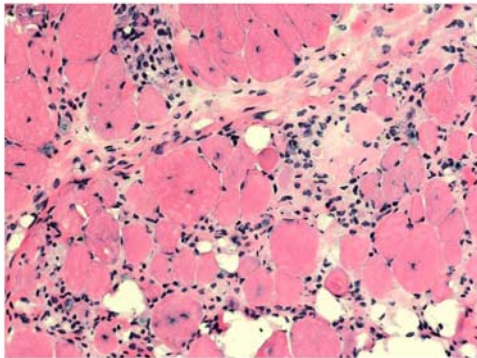

GA

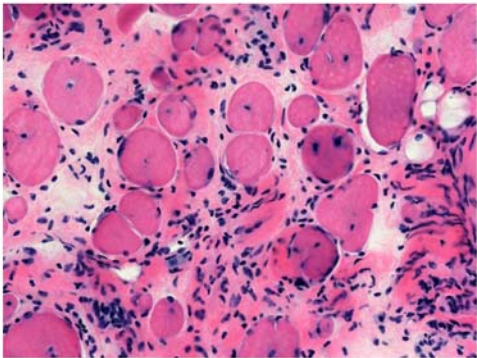

DIA

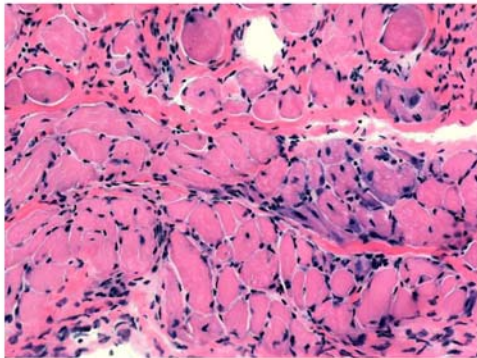

TA

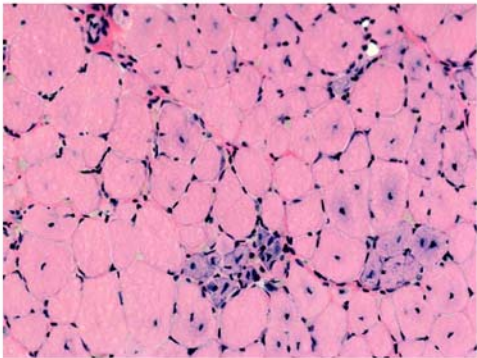

IP

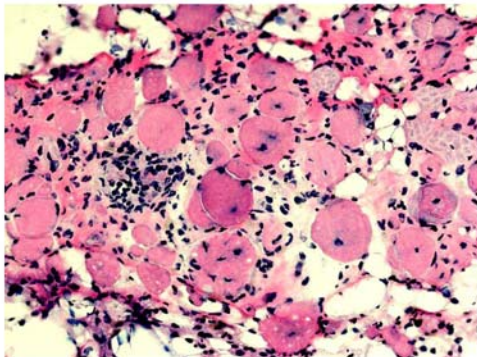

HS

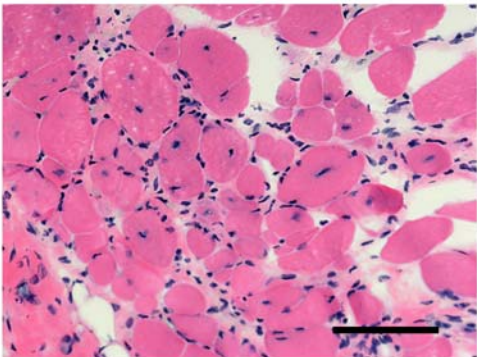

Supplement: Additional file 2 — Figure S2 Histopathology analyses of various muscles from dystrophin/dysferlin double-knockout mice. H & E-stained muscle sections of triceps (TC), gastrocnemius (GA), diaphragm (DIA), tibialis anterior (TA), iliopsoas (IP), hamstring (HS) and gluteus (GT) muscles from dystrophin/dysferlin double-knockout (DKO) mice at one and one-half years of age. Scale bar: 100 μm. [file 2044-5040-1-35-S2.PDF]
